# Supplementary material for: Enhancing machine learning performance in cardiac surgery ICU: Hyperparameter optimization with metaheuristic algorithm
Source: PLoS One. 2025 Feb 10;20(2):e0311250. doi: 10.1371/journal.pone.0311250 (PMC11809875; doi:10.1371/journal.pone.0311250)
Supplement: Attachment — Submitted filename: Response to Reviewers.docx [file pone.0311250.s001.docx]

**Rebuttal Letter**

30 June 2024

**Journal:** PLOS ONE

Manuscript Title: Enhancing Machine Learning Performance in Cardiac Surgery ICU: Hyperparameter Optimization with Metaheuristic Algorithm

Submission ID: PONE-D-24-07915

### **Dear Prof. Xin Gao,**

It is our pleasure to submit the revision of our manuscript entitled: “Enhancing Machine Learning Performance in Cardiac Surgery ICU: Hyperparameter Optimization with Metaheuristic Algorithm” to be considered for publication in PLOS ONE. We deeply appreciate the opportunity to enhance our work based on the valuable feedback provided.

Below, the point-by-point response to the comments of reviewers will be found. All modifications are highlighted in the revised manuscript for ease of the review process.

We sincerely would like to thank you and the reviewers for your insightful and constructive comments.

**Best wishes and kind regards,**

**Rouzbeh Ghousi**

**School of Industrial Engineering**

**Iran University of Science and Technology**

**Tehran**

**Iran**

The followings are our point-by-point responses to the reviewers’ comments:

# Comments from editor:

Thank you for submitting your manuscript to PLOS ONE. We appreciate the effort you have put into this research and the potential impact it can have on ICU ventilator management. After carefully reviewing the feedback from the reviewers, I have decided that a major revision is necessary before we can consider your manuscript for publication.

Given the mixed reviews, I have decided that a major revision is necessary. Please address the following key points to improve your manuscript:

- Provide clear explanations for your choice of models (CatBoost, LDA, ANN) and the use of simulated annealing over other methods such as cross-validation or grid search. Additionally, ensure that the rationale behind your hyperparameter tuning ranges is well-articulated.

***Response****:*

Thank you for your detailed assessment and constructive feedback. We appreciate the opportunity to clarify and expand on the key aspects of our manuscript as highlighted.

- **Choice of Models:** Our selection of CatBoost, Linear Discriminant Analysis (LDA), and Artificial Neural Networks (ANN) was driven by their unique capabilities and compatibility with the specific challenges of our dataset and study objectives. CatBoost excels in handling categorical data and delivers efficient performance in both regression and classification tasks without extensive preprocessing. LDA was selected for its proficiency in reducing dimensionality and enhancing class separation, crucial for the complex data structures typical in medical datasets. ANN was chosen for its robustness in modeling non-linear relationships, which are prevalent in medical data. Additionally, the inclusion of XGBoost, recommended by the TPOT AutoML algorithm, complements the ensemble by enhancing its predictive accuracy and robustness, leveraging its gradient boosting framework. Also, these models were validated against empirical evidence, ensuring their superior performance in metrics such as Accuracy, Precision, Sensitivity, Specificity, and F1-score, as documented in [26] reference of revised manuscript. Additionally, we reviewed related studies to these models, as presented in Table 1, to support our model selection.
- **Use of Simulated Annealing (SA):** We employed SA and Genetic Algorithm (GA) for hyperparameter tuning instead of traditional methods like grid search or cross-validation due to their superior efficiency in navigating complex, high-dimensional parameter spaces. These metaheuristic algorithms offer a significant advantage in avoiding local optima and exploring the solution space more globally, thus providing optimal model performance. Moreover, SA and GA allow dynamic hyperparameter adjustments which are crucial for deep learning models and complex architectures, enhancing the model's adaptability and reducing the risk of overfitting. This approach not only aligns with cutting-edge practices in machine learning but also ensures that our models perform robustly on unseen datasets.
- **Hyperparameter Tuning Ranges:** The selection of hyperparameter ranges was meticulously considered, drawing on a blend of foundational guidelines from model developers documentations, expert recommendations tailored to our research context, and empirical evidence from related studies that referenced in Table 3 of revised manuscript. This methodical approach ensures that our hyperparameters are not only theoretically justified but also pragmatically refined to meet the specific requirements of our dataset, enhancing both the performance and reliability of our models.

These updates and justifications are thoroughly documented in the revised manuscript to enhance transparency and understanding of our methodological choices, ensuring that our study remains relevant to practical applications in healthcare settings.

We hope these revisions and explanations address your concerns and substantially strengthen our manuscript. We look forward to your feedback and are eager to make further modifications if required. Thank you for your guidance and consideration.

Locations of Changes in the Revised Manuscript:

- Update: Introduction (Section 1, paragraph 11, page 4)
- Update: Machine Learning & Metaheuristic Models (Section 2.3.1, paragraph 2 and 4, page 11)
- Reconsider the approach of removing features with more than 15% missing data. Explore and discuss imputation methods and assess the importance of these features to ensure they are not prematurely discarded.

***Response****:*

Thank you for your insightful comments regarding our initial handling of missing data. We recognize the potential value of features with more than 15% missing data and have revised our approach to ensure no informative features are prematurely discarded.

In response to your comment, we have adopted the K-Nearest Neighbors (KNN) imputation method to manage missing values, particularly in the PH, MG, and MAP columns. This method was chosen for its effectiveness in maintaining the integrity of the underlying data structure by imputing missing values based on the similarity of observable data points. This approach helps ensure that we retain potentially predictive information which might have been lost with straightforward removal.

Further, we conducted a rigorous feature importance analysis using Random Forest (RF) and Gradient Boosting Machine (GBM) models to reassess the significance of all features, including those initially identified with higher missing data rates. By setting a thoughtful importance score threshold of 0.001, we ensured that our model includes only those features that contribute significantly to predictive accuracy and generalizability. This iterative process of testing and evaluation has allowed us to refine our feature set effectively, ensuring that all retained features are justified in their inclusion based on their predictive power.

These methodological refinements are detailed in the revised sections of our manuscript to enhance the clarity and robustness of our data handling strategies:

Locations of Changes in the Revised Manuscript:

- Update: Introduction (Section 1, page 4).
- Update: Preprocessing (Section 2.2, page 8).
- Update: Handling Missing Values and Applying Feature Importance (Section 2.2.1, pages 8 and 9).
- Add: Figure 2
- Update: Table 1
- Update: Table 2

We hope these updates address your concerns effectively, demonstrating our commitment to a thorough and careful analysis. We are confident that these changes have strengthened our manuscript, making the findings more robust and broadly applicable. Thank you again for your guidance, and we look forward to any further suggestions you may have.

- Validate your model across multiple independent datasets to enhance its robustness and generalizability. Address potential overfitting issues, particularly with ANN, and consider the use of ensemble models for improved predictive performance.

***Response:***

Thank you for your insightful feedback and for emphasizing the critical need for robust model validation across multiple datasets, as well as the importance of addressing overfitting issues, particularly with Artificial Neural Networks (ANN).

- **Model Validation Across Multiple Datasets:** We acknowledge the limitations posed by using a dataset from a single hospital and understand the necessity for broader validation to ensure our model's general applicability and robustness. While our dataset provided a solid foundation for developing and testing our predictive models, we agree that expanding our dataset to include multiple independent sources would significantly enhance the generalizability of our findings. Given the challenges associated with acquiring comparable datasets due to privacy concerns and data-sharing restrictions, we have implemented a rigorous 10-fold cross-validation method as an interim solution to simulate external validation. This method allows us to rigorously assess model performance across different subsets of our data, minimizing the bias and variance and providing a robust evaluation framework. Additionally, to further validate our models and ensure robust testing against new, unseen data, we have partitioned our dataset into three distinct parts: training, validation, and testing.
- **Addressing Overfitting in ANN:** We have taken specific steps to mitigate the risk of overfitting, particularly in our ANN model. These steps include implementing regularization techniques such as L1 and L2 regularization, which penalize large weights in the ANN, LDA, CatBoost, and XGBoost model’s architecture, effectively reducing the model's complexity and preventing it from fitting too closely to the training data. Furthermore, we utilized a weighted voting ensemble model that integrates predictions from multiple models, including CatBoost, LDA, ANN, and the newly added XGBoost. This ensemble approach leverages the strength of each model, balancing out their individual weaknesses, and has demonstrated improved predictive performance and robustness. The ensemble's weights were optimized using a grid search to maximize the F1-score on the validation set, which has shown significant enhancements in sensitivity and specificity, crucial for clinical settings.
- **Ensemble Models for Improved Predictive Performance:** By integrating these models into an weighted voting ensemble model, we effectively combine their predictive capabilities to produce a more accurate and stable model. This strategy not only helps in mitigating individual model biases and variances but also enhances the overall predictive power of our approach, making it more reliable for practical application in ICU settings.

We appreciate the opportunity to enhance our manuscript based on your valuable insights. The modifications we have made, detailed in the updated sections of the manuscript, underscore our commitment to rigorous scientific standards and our dedication to advancing healthcare analytics. We believe these changes address your concerns and substantially strengthen the paper.

Locations of Changes in the Revised Manuscript:

- Update: Introduction (Section 1, page 4)
- Update: Preprocessing (Section 2.2, page 8)
- Update: Handling Missing Values & Applying Feature Importance (Section 2.2.1, pages 8 and 9)
- Update: Machine Learning & Metaheuristic Models (Section 2.3.1, paragraphs 4 and 5, pages 11 and 12)
- Add: Ensemble Model Construction (Section 2.3.2, pages 14 and 15).
- Update: Evaluation (Section 2.3.4, paragraph 1, pages 17 and 18)
- Update: Results and discussion (Section 3)

We are grateful for your guidance and are prepared to make further revisions as needed to meet the journal’s standards. Thank you once again for your constructive suggestions.

- (Optional) Include comparisons of simulated annealing with other optimization algorithms and techniques such as AutoML. This will help demonstrate the advantages and limitations of your approach.

***Response:***

Thank you for suggesting the comparison of simulated annealing (SA) with other optimization algorithms. In response, we have implemented the Genetic Algorithm (GA) to tune the hyperparameters of our base classifiers, including CatBoost, LDA, ANN, and XGBoost. We then compared their performance on the final prediction on the test dataset. Furthermore, we employed AutoML to assess its performance and compared its results with the more effective of the two algorithms, SA or GA. This approach allowed us to directly evaluate the performance and efficacy of AutoML in relation to our more manually intensive, customized tuning processes.

The primary advantage of using both SA and GA in our study is the ability to have granular control over the tuning process, which is particularly beneficial for complex datasets and model configurations. These metaheuristic algorithms provide flexibility in exploring the hyperparameter space that AutoML may not offer, especially when dealing with specific constraints or preferences that are tailored to our research objectives. Furthermore, our predefined range of hyperparameters is broader than that typically utilized by TPOT AutoML, allowing for more extensive exploration and optimization. Conversely, AutoML offers a more streamlined and automated approach, reducing the need for detailed algorithmic knowledge and manual configuration. This makes AutoML highly accessible and efficient, particularly for general applications where extensive customization is not required. However, it can sometimes result in a lack of transparency in the model training process, which can be a limitation for research purposes where understanding the influence of each hyperparameter is crucial.

In the revised manuscript, we have highlighted these comparisons, detailing the pros and cons of using SA and GA versus AutoML frameworks. This includes discussions on the trade-offs between control and convenience, as well as the impact of each method on the overall performance and interpretability of our models. We believe this comprehensive analysis will provide readers with valuable insights into the appropriate contexts and conditions for the use of these different hyperparameter tuning approaches.

Locations of Changes Related to this Comment in the Revised Manuscript:

- Update: Abstract.
- Add: Comparison (Section 2.3.3, paragraphs 2, 3, and 4).
- Update: Results and discussion (Section 3, paragraphs 6).
- Update: Results and discussion (Section 3).
- Add: Table 7
- Discuss the clinical significance of your findings in more detail and incorporate aspects of model explainability to improve trust and adoption in clinical settings. Highlight how your model integrates with existing scoring systems and its potential impact on patient care.

***Response:***

Thank you for your insightful feedback and the opportunity to enhance the clinical relevance and explainability of our predictive models.

- **Clinical Significance and Explainability:** In response to your suggestion, we have expanded the discussion of the clinical significance of our findings within the revised manuscript, emphasizing not only the improved predictive accuracy of our models but also their potential impact on patient care and healthcare resource management. We have provided a detailed explanation of how our models can improve decision-making in intensive care unit (ICU) environments, particularly in the efficient allocation of ventilators, which is crucial in critical care settings. To address the need for model explainability, which is vital for user trust and adoption in clinical settings, we have incorporated advanced techniques for interpreting our models. This includes the use of feature importance analysis from Random Forest (RF) and Gradient Boosting Machine (GBM) models. By identifying and highlighting the most influential features, we ensure that healthcare professionals can understand the rationale behind the model's predictions.
- **Integration with Existing Scoring Systems:** Regarding the integration of our model with established clinical scoring systems such as APACHE and SAPS, we have carefully considered this suggestion. However, our analysis and literature review indicate that these scoring systems, while effective for mortality prediction in ICU settings, do not align directly with the specific objectives of ventilator need prediction. Our model is designed to complement, rather than replace, existing frameworks by providing targeted predictions about ventilator requirements, thereby filling a critical gap in ICU resource management. While the integration of our model with traditional scoring systems is not directly feasible due to differing objectives and metrics, we propose that our model could serve as an adjunct tool that enhances the existing protocols. By leveraging our predictive model, healthcare providers can gain additional insights into ventilator needs, which can be integrated with broader clinical assessments to enhance patient care.

Locations of Changes Related to this Comment in the Revised Manuscript:

- Update: Handling Missing Values and Applying Feature Importance (Section 2.2.1, page 8).
- Update: Results and discussion (Section 3, last paragraph).

We believe these revisions will significantly strengthen the manuscript by highlighting the practical utility and scientific rigor of our approach. We are grateful for the opportunity to refine our work based on your valuable feedback and look forward to further suggestions. Thank you once again for your constructive comments.

- Ensure that all figures and plots in the manuscript are of high quality and clearly legible.

***Response:***

Thank you for your feedback on enhancing the visual elements of our manuscript. We have replaced the blurred plots with high-resolution versions, each exported at a minimum of 600 DPI. This ensures that all plots are now clear and legible.

- Please provide a detailed response to the reviewers' and editor's comments and submit a revised version of your manuscript along with a point-by-point response letter. The revised manuscript will undergo another round of review to ensure the concerns have been adequately addressed.

***Response:***

Thank you for your detailed feedback and the opportunity to refine our manuscript. We have carefully considered each comment from the reviewers and the editor and have made comprehensive revisions accordingly. A revised version of the manuscript has been submitted, accompanied by a detailed point-by-point response letter addressing each comment. In this response letter, we outline the changes made to the manuscript. Each modification in the manuscript is clearly highlighted to facilitate the review process.

We trust that these revisions meet the concerns raised during the initial review and enhance the manuscript's contribution to the field. We look forward to the subsequent round of review and are committed to making further adjustments as recommended to ensure the manuscript fully meets the journal's standards.

# Comments from Reviewer 1:

It is a well-designed and executed study that addresses an important challenge in the healthcare domain. The combination of ML models with SA for hyperparameter optimization is an effective approach that has not been widely explored in the context of cardiac surgery ICU ventilator prediction. The enhancement in the predictive performance, particularly the increase in sensitivity (recall), after applying the Simulated Annealing algorithm for hyperparameter tuning is a noteworthy finding that can have direct implications for patient care and resource allocation in the ICU setting.

- To further strengthen your manuscript, I would suggest considering discussing the clinical significance of your findings in more detail, highlighting the potential impact on patient care and healthcare resource management.

***Response:***

Thank you for your thoughtful feedback and the positive comments on our study's design and execution. We highly value your comments to enhance our study.

In response, we acknowledge the significance of connecting our research findings to relevant clinical applications and have revised our manuscript accordingly. In the results and discussion section, we present a comprehensive examination of the clinical significance of our findings, highlighting the enhanced predictive powers of our models and their potential impact on patient care and healthcare resource management. We investigate the capacity of findings and developed model to improve decision-making procedures in intensive care unit (ICU) environments, thereby optimizing the assignment of ventilators and enhancing outcomes for patients in critical care.

Locations of Changes in the Revised Manuscript:

- Update: Results and discussion (Section 3).
- Also acknowledge the limitations of your study, such as the single-center nature of the dataset, and discuss the need for external validation of the proposed models.

***Response:***

We truly value your insightful comment, which highlights the importance of discussing the limitations of our study. We acknowledge that our research's dataset originates from a single hospital, potentially affecting the scope of our findings. In the revised "Results and Discussion" section of our manuscript, we have explicitly addressed this limitation. We emphasize the importance of incorporating datasets gathered from multiple hospitals to externally validate our models in future research. Additionally, we are actively working to gather more data to broaden the scope and improve the robustness of our future studies.

Locations of Changes in the Revised Manuscript:

- Update: Results and discussion (Section 3, paragraph 9, page 22).
- Lastly, explore the possibility of integrating your hybrid model with existing scoring systems (e.g., APACHE, SAPS) to further enhance the predictive capabilities and provide a more comprehensive assessment of patient risk.

***Response****:*

Thank you for your suggestion to explore the integration of our model with scoring systems such as APACHE and SAPS. As discussed in the literature review in the Introduction section, these scoring models are predominantly utilized for predicting mortality in ICU settings, as referenced in [3], [4], [5], [6], [7], and [14] in the revised manuscript. Our study, however, focuses on predicting the need for ventilatory support rather than mortality. The objectives and underlying metrics of mortality prediction models differ significantly from those required for ventilator need prediction, making a direct integration of such systems with our model fundamentally unfitting.

Instead, our approach leverages machine learning models tailored specifically to predict ventilator requirements. To enhance the precision and effectiveness of these predictions, we have employed metaheuristic methods that optimize the performance of our ensemble model. This strategy has proven effective, as evidenced by the significant results achieved in our analyses. Therefore, while the integration of traditional scoring systems presents an interesting avenue for broadening model capabilities, it does not align with the specific goals and methodological framework of our current research.

# Comments from Reviewer 2:

In the paper titled "Enhancing Machine Learning Performance in Cardiac Surgery ICU: Hyperparameter Optimization with Metaheuristic Algorithm," the authors present a significant advancement in predictive healthcare analytics. The study addresses the critical need for ventilator allocation in cardiac surgery ICUs, leveraging a hybrid approach that combines machine learning (ML) and deep learning (DL) models with a metaheuristic algorithm.

One of the key strengths of this study lies in its innovative methodology, which employs Simulated Annealing (SA) for hyperparameter tuning, significantly improving the prediction performance of ML and DL models. Notably, the study demonstrates a remarkable 14% increase in sensitivity for Artificial Neural Networks (ANN) after applying SA.

Furthermore, the authors provide comprehensive insights into the challenges of ICU care, particularly in ventilator management, and highlight the potential of ML techniques to address these challenges effectively. By leveraging a rich dataset obtained from a hospital, the study offers practical implications for optimizing ventilator allocation in critical healthcare settings.

The paper's contribution extends beyond methodology, as it systematically addresses the gap in predicting ventilator needs specifically for cardiac surgery ICU patients. This focus is particularly relevant during pandemics or periods of increased ICU admissions, where efficient resource allocation can be lifesaving.

Overall, the study provides a well-researched and meticulously executed analysis that not only enhances our understanding of ventilator management in cardiac surgery ICUs but also underscores the potential of ML techniques, especially when integrated with metaheuristic algorithms, to improve patient care outcomes in critical healthcare scenarios.

***Response****:*

Thank you very much for your positive and encouraging feedback on our manuscript. We greatly appreciate your recognition of the innovative methodology and the practical implications of our study. We have carefully considered all reviewers' comments and have made improvements to our manuscript to enhance its clarity and impact.

Your acknowledgment of our work's strengths is greatly appreciated and has motivated us to refine our manuscript further. Thank you once again for your valuable insights and supportive evaluation.

# Comments from Reviewer 3:

Thanks for your contribution. I have couple questions below:

**Technical:**

- The paper conducted PCA but the feature space is not large - 32 features and PCA can throw unsatisfying results. Normally L1, L2 regularization can alleviate overfitting issue, all of the LDA/Catboost/ANN have ways to do that.

***Response****:*

Thank you for your time and the insightful questions that have significantly contributed to improving our work.

Your feedback regarding the utilization of PCA in our study is greatly appreciated. After a careful review and consideration of the insights from your feedback, we decided to remove PCA from our methodology. This allows us to maintain the interpretability of all original features without the risk of losing information through transformation.

Instead of using PCA, we focused on feature importance assessments using algorithms like Random Forest (RF) and Gradient Boosting (GBM). This approach allowed us to identify the most informative features directly influencing our predictive models, optimizing model performance without the abstraction introduced by PCA. After implementing RF and GBM, we select the top 17 features that have the most influence on the target variable.

As you stated, to address potential overfitting concerns, we have implemented specific regularization techniques in our models:

- We used a shrinkage parameter akin to regularization in **LDA**, which helps control model complexity. To find the optimal value for this parameter, we tune it using SA and GA. Both algorithms found 0.6 for shrinkage.
- **CatBoost** regulates model complexity not through L1 and L2, but through parameters such as l2_leaf_reg, which we set at 3, which functions similarly to L2 regularization. Setting l2_leaf_reg at 3 was determined to provide a balance between model flexibility and the risk of overfitting, based on preliminary testing and validation on our dataset. This value helps in smoothing the learning process, ensuring that the model generalizes well on unseen data while maintaining sufficient complexity to capture underlying patterns in the data.
- **XGBoost** utilizes both L1 and L2 regularizations, with alpha set at 0.01 for L1 and lambda set at 1.0 for L2. We set the alpha parameter at 0.01 for L1 regularization to promote feature selection by reducing less important feature weights to zero, and the lambda parameter at 1.0 for L2 regularization to penalize large weights and prevent overfitting, ensuring the model’s generalization across diverse datasets.
- In the **ANN**, we apply L1 and L2 regularizations directly to the layers, with kernel_regularizer parameters set as L2 (0.01) and L1 (0.01), and a combined L1_L2 regularization with L1=0.01 and L2=0.01. We set the L1 and L2 regularization values at 0.01 in the ANN model to strike a balance between reducing model complexity and maintaining predictive power. This specific value helps in mitigating overfitting by penalizing large weights without excessively compromising the model’s ability to learn from complex data patterns. The choice of 0.01 for both L1 and L2 regularizations was determined through iterative testing to optimize the model's performance on validation data, ensuring it generalizes well on unseen datasets while maintaining a robust feature selection mechanism.

These regularization strategies are effective at managing overfitting and ensuring the robustness of our models.

By omitting PCA, we retain full control over our feature set and avoid the possible drawbacks of dimensionality reduction, such as the loss of meaningful variance or misinterpretation of transformed features. This decision, along with our robust regularization and feature selection strategies, is fully detailed and justified in the revised manuscript to ensure transparency and clarity in our methodological choices.

Locations of Changes in the Revised Manuscript:

- Update: Introduction (Section 1, page 4).
- Update: Preprocessing (Section 2.2, page 8).
- Update: Handling Missing Values and Applying Feature Importance (Section 2.2.1, paragraph 3, page 8).
- Update: Machine Learning & Metaheuristic Models (Section 2.3.1, paragraph 5, page 11).
- Update: Table 1
- Remove: Utilizing PCA For Feature Selection (Old Section 2.2.5)
- Do have concern over removing missing feature > 15%. Some may be important and useful in prediction. Have we tried any imputation method for missing values. Probably looking at feature importance to see if some missing features are badly removed.

***Response****:*

Thank you for your feedback regarding our initial approach to handling missing data and feature selection. In response, we have refined our preprocessing strategy to ensure a more robust analysis, which is thoroughly detailed in the revised manuscript.

To address the concerns over the removal of features with more than 15% missing data, we employed the K-Nearest Neighbors (KNN) imputation method to handle missing values, in the PH, MG, and MAP columns. This method helped us preserve the underlying data structure and maintain feature integrity by considering the similarity between data points, ensuring that no potentially predictive information was lost.

Additionally, we conducted a feature importance analysis using both Random Forest (RF) and Gradient Boosting (GBM) models. This analysis helped us to identify and retain the most informative features, using a threshold importance score of 0.001. The threshold was set after iterative testing and evaluation to determine the optimal cutoff that maximizes model performance and generalizability across different tests. We ensured that only features with significant predictive power were included in further analyses. The intersection of important features identified by these models was carefully considered to ensure robustness in our feature set.

Locations of Changes in the Revised Manuscript:

- Update: Introduction (Section 1, page 4).
- Update: Preprocessing (Section 2.2, page 8).
- Update: Handling Missing Values and Applying Feature Importance (Section 2.2.1, pages 8 and 9).
- Add: Figure 2
- Update: Table 1
- Update: Table 2
- The paper experimented with CatBoost/LDA/ANN but didn't give articulate the reasons behind it.

***Response****:*

Thank you for your valuable feedback. We appreciate the opportunity to clarify the rationale behind our choice of CatBoost, Linear Discriminant Analysis (LDA), and Artificial Neural Networks (ANN) for our research. We have updated our manuscript to provide a clearer rationale for selecting these models.

In our selection process, we considered each model's unique capabilities and their compatibility with the specific requirements of our dataset and research objectives. We chose CatBoost for its ability to handle categorical features without extensive preprocessing and its efficiency in both regression and classification tasks. LDA was selected for its strength in dimensionality reduction and class distinction based on linear combinations of features, which is vital for medical datasets. ANN was chosen for its capability to model non-linear relationships in medical data through deep learning architectures. Also, XGBoost was included due to its proven efficacy in numerous studies and its recommendation by the TPOT AutoML algorithm, enhancing the ensemble's performance. These models were validated against empirical evidence, ensuring their superior performance in metrics such as Accuracy, Precision, Sensitivity, Specificity, and F1-score, as documented in [26] study.

Additionally, we reviewed related studies, as presented in Table 1 of the manuscript, to support our model selection. This ensures that each model contributes uniquely to the ensemble, enhancing the overall predictive power. The integration of XGBoost into our ensemble model provides a robust platform for comparative analysis against other classifiers, leveraging each model's strengths to enhance the ensemble's overall performance. This strategic choice is detailed in the revised manuscript, which explains the selection process and the reasons these models are well-suited for our specific data characteristics and research goals.

Locations of Changes in the Revised Manuscript:

- Update: Machine Learning & Metaheuristic Models (Section 2.3.1, paragraph 2, page 11).
- The paper didn't articulate the reasons using SA to conduct hyper-parameter tunings although its valid theoretically. Other methods, e.g. Cross-validation can do the tuning easily.

***Response****:*

Thank you for your feedback and the opportunity to clarify the choice of using Simulated Annealing (SA) for hyperparameter tuning in our study.

We opted for metaheuristic algorithms such as SA and the Genetic Algorithm (GA) over traditional methods like cross-validation, grid search, and random search due to their superior efficiency in navigating complex and high-dimensional parameter spaces. These algorithms excel at finding optimal solutions more efficiently than exhaustive search methods such as grid search, which becomes computationally impractical as the number of hyperparameters increases. Unlike grid search, metaheuristics can escape local optima and explore the solution space more globally, which is crucial for achieving optimal model performance.

Metaheuristic algorithms are highly scalable and adaptable to various optimization problems without significant modifications, making them suitable for different models and datasets. This flexibility enhances their utility across diverse machine learning tasks. Moreover, SA and GA are particularly adept at handling non-linear and non-convex optimization challenges where traditional methods might fail to find the global optimum. These algorithms use probabilistic and evolutionary principles, respectively, to navigate through complex search landscapes effectively.

Another significant advantage of metaheuristic algorithms is their reduced risk of overfitting. By intelligently exploring the search space and avoiding overfitting traps that typically ensnare more rigid methods like grid search, these algorithms can identify hyperparameters that generalize better to unseen data. This capability is particularly important in applications where model performance on new, unseen datasets is critical.

Furthermore, metaheuristics can be seamlessly integrated into complex model architectures, offering dynamic hyperparameter adjustments based on the model's learning progress. This feature is especially beneficial in deep learning models, where traditional tuning methods such as cross-validation, used in conjunction with grid or random search, assume static parameter sets and do not adjust to changes during the training process.

Overall, the strategic advantages of metaheuristic algorithms—in terms of efficiency, flexibility, and effectiveness in managing complex optimization problems—make them a preferred choice for hyperparameter tuning in advanced machine learning applications. They provide robust solutions that enhance model accuracy and reliability, proving essential in cutting-edge research and practical scenarios where quick, effective model tuning is required. This approach ensures that our methodology remains at the forefront of technological advancements and applicable to real-world scenarios.

Locations of Changes in the Revised Manuscript:

- Update: Introduction (Section 1, paragraph 11, page 4).
- Have we checked overfitting issues when evaluation the models, ANN can be easily overfitted.

***Response****:*

Thank you for highlighting the critical issue of overfitting, particularly concerning Artificial Neural Networks (ANN). In our study, we have taken several proactive measures to prevent overfitting and ensure that our models generalize well to new, unseen data. These measures include:

1. **Regularization Techniques:** We implemented L1 and L2 regularization to add penalties on the magnitude of the weights in the loss function, effectively constraining them and reducing overfitting. This helps in keeping the model weights small, making the network simpler and less likely to fit noise in the training data. We apply L1 and L2 regularizations directly to the layers, with kernel_regularizer parameters set as L2 (0.01) and L1 (0.01), and a combined L1_L2 regularization with L1=0.01 and L2=0.01.
2. **Cross-Validation:** We employed 10-fold cross-validation to validate the stability and reliability of the model across different data splits. This method helps in ensuring that the model’s performance is consistent and not just tailored to a specific subset of data.
3. **Hyperparameter Tuning:** We carefully tuned hyperparameters using metaheuristic algorithms such as Simulated Annealing (SA) and Genetic Algorithms (GA) to find optimal settings that enhance model performance while preventing overfitting. This approach allowed us to efficiently navigate the high-dimensional parameter spaces and escape local optima.
4. **Ensemble Model:** By combining the predictions from multiple models, we improved the robustness and generalization of our predictions. This approach helps in reducing the variance and bias of the model predictions.

These strategies collectively enhance the robustness of our ANN and other models, making them more reliable and effective in predicting outcomes on new data. We detailed these methodologies in the manuscript to ensure transparency and provide insights into our rigorous model validation processes.

Locations of Changes in the Revised Manuscript:

- Update: Machine Learning & Metaheuristic Models (Section 2.3.1, paragraph 5, page 11).
- For predictive purpose, it may be benefitted from an ensembled model.

***Response****:*

Thank you for this great proposal that greatly improves our results. Based on your valuable feedback, we refined our methodology to incorporate an ensemble model that integrates the predictive capabilities of CatBoost, LDA, ANN, and the newly added XGBoost. This diverse ensemble leverages each model’s strengths through a weighted voting strategy.

Each base classifier—CatBoost, LDA, ANN, and XGBoost—was first independently trained on a balanced dataset using the SMOTE technique to ensure equal representation of each class, which is crucial for eliminating bias towards the majority class in our predictions. After training, the performance metrics of each model were evaluated.

The ensemble model employs a dynamic weighting system where the contributions of each model's predictions are weighted differently based on their performance metrics such as accuracy, precision, sensitivity, and F1-score. These weights are adjusted to maximize the overall performance of the ensemble on the validation set, focusing on achieving the highest F1-score. This score is particularly important as it balances both precision and sensitivity, ensuring that our model performs reliably under different clinical conditions.

This method of using a weighted voting strategy allows us to harness the unique strengths of each individual classifier and mitigate their weaknesses, leading to improved prediction accuracy and robustness of the overall model. The ensemble approach has demonstrated superior performance compared to the individual models, highlighting the efficacy of combining multiple predictive techniques in an integrated framework.

Locations of Changes in the Revised Manuscript:

- Update: Abstract
- Update: Introduction (Section 1, paragraph 12, page 4).
- Update: Table 1
- Update: Modeling (Section 2.3, page 10)
- Update: Machine Learning & Metaheuristic Models (Section 2.3.1, paragraphs 1, 2, 3, and 8, pages 11, 12, and 13)
- Add: Ensemble Model Construction (Section 2.3.2, pages 14 and 15).
- Add: Comparison (Section 2.3.3, pages 15, 16, and 17).
- Update: Evaluation (Section 2.3.4, pages 17 and 18).
- Update: Results and discussion (Section 3).
- Update: Table 4
- Update: Table 6
- Add: Table 7
- The paper lacks comparison of SA to other optimization algorithm.

***Response****:*

Thank you for your constructive feedback, which has significantly shaped the revisions in our manuscript. Following your insightful comment, we have incorporated the Genetic Algorithm (GA) to optimize the hyperparameters of our base classifiers, including CatBoost, LDA, ANN, and XGBoost, which was added to our study in the revised manuscript.

We then conducted a comparative analysis of the results obtained using Genetic Algorithm (GA) with those obtained from Simulated Annealing (SA) and AutoML. This comprehensive comparison has allowed us to refine our methodology further and provide a robust evaluation of these optimization algorithms. The results of this comparison are detailed in the revised manuscript. This enhancement not only addresses your concern but also significantly bolsters the scientific rigor of our research.

Locations of Changes in the Revised Manuscript:

- Update: Abstract
- Update: Introduction (Section 1, paragraph 11, page 4).
- Update: Machine Learning & Metaheuristic Models (Section 2.3.1, paragraphs 5, page 11)
- Add: Comparison (Section 2.3.3, pages 15, 16, and 17).
- Add: Algorithm 2 (Section 2.3.3, page 15).
- Update: Evaluation (Section 2.3.4, page 18).
- Update: Results and discussion (Section 3).
- Update: Table 5
- Update: Table 6
- Add: Table 7

**Publication:**

- The plots in the end seem blurred.

***Response****:*

Thank you for your feedback regarding the plots quality. We have replaced the blurred plots with high-resolution versions, each exported at a minimum of 600 DPI. This ensures that all plots are now clear and legible.

# Comments from Reviewer 4:

This study introduces a novel approach to predict the need for ventilators in cardiac surgery ICU patients by integrating machine learning (ML) and deep learning (DL) models with a metaheuristic algorithm, specifically Simulated Annealing (SA), for hyperparameter optimization. The research is highly relevant and valuable in reality, given the critical importance of ventilator allocation in healthcare settings, especially during the pandemic.

**Strengths:**

- The paper effectively combines various ML and DL techniques with a metaheuristic optimization algorithm, which is innovative in the field of medical data.

***Response****:*

Thank you very much for your positive and encouraging feedback on our manuscript. We greatly appreciate your recognition of the innovative methodology that combines various machine learning and deep learning techniques with a metaheuristic optimization algorithm. We believe that this integration is crucial for advancing predictive analytics in the medical field, especially given the complex nature of medical data and the critical need for accurate predictions in healthcare settings. Your feedback motivates us to continue refining our approach and exploring further applications of these advanced techniques in medical research.

- The topic is of high relevance, potentially offering significant improvements in ICU resource management and patient care.

***Response****:*

Thank you for your positive feedback and for recognizing the relevance of our study. We are encouraged by your acknowledgment of the potential impact our research can have on ICU resource management and patient care. This validation supports our aim to contribute meaningful advancements in healthcare practices, particularly in optimizing the allocation of critical resources and enhancing patient outcomes in intensive care settings. We appreciate your support and are motivated to pursue further research in this vital area.

**Weakness:**

- The study is limited to data from a single hospital source and the data access is restricted. Validation of the model across multiple independent datasets would enhance the robustness and general applicability of the findings.

***Response****:*

Thank you for highlighting the limitations concerning the dataset used in our study. We acknowledge that our data, being sourced from a single hospital, may limit the generalizability of our findings. This issue is acknowledged in the revised manuscript, particularly in the results and discussion section. We discuss the imperative need for future research to include datasets gathered from multiple hospitals to externally validate our models. This strategy will undoubtedly enhance the robustness and applicability of our predictive models across various clinical settings, ensuring that our findings can be reliably used to inform broader healthcare practices.

Furthermore, it is important to note the inherent challenges in obtaining external datasets in this area, primarily due to legal restrictions and privacy concerns. Hospitals and healthcare centers typically do not share this type of data, which adds complexity to conducting broad-based, multi-center studies. Despite these challenges, we have endeavored to establish a precise and rigorous methodology to validate our model internally. This includes dividing the data into three distinct parts: training, testing, and validation. Additionally, we have implemented a 10-fold cross-validation procedure and employed an ensemble learning algorithm to further prove the validity, robustness, and generality of implemented models. These methodologies are designed to compensate for the lack of external validation and ensure that our results are as reliable and applicable as possible under the given circumstances.

Locations of Changes in the Revised Manuscript:

- Update: Evaluation (Section 2.3.4, paragraph 1, pages 17 and 18).
- Update: Results and discussion (Section 3, page 22).
- There is insufficient discussion regarding the limitations of the applied models and the potential biases within the study. A thorough exploration of these aspects would provide a more comprehensive understanding of the model's utility and constraints.

***Response****:*

Thank you for your insightful feedback. We appreciate your emphasis on the importance of discussing the limitations of the applied models and addressing potential biases within our study. In the revised manuscript, we have expanded our discussion of these crucial aspects in the results and discussion section to provide a more comprehensive understanding of the model’s utility and constraints.

One significant limitation that we acknowledge is the use of a dataset from a single center, which may affect the generalizability of our findings. To mitigate this issue, we implemented robust data partitioning strategies and utilized 10-fold cross-validation. Additionally, we employed an ensemble method that combines the strengths of multiple models through a weighted voting approach, which helps improve the stability and accuracy of our predictions. This ensemble approach mitigates the limitations of each individual machine learning model, reducing the risk of overfitting and bias that might arise from the dataset's specific characteristics.

Furthermore, in the revised manuscript, we have explicitly addressed potential biases and outlined additional strategies that could be employed in future studies to enhance the models’ applicability to other settings. This includes suggestions for multi-center studies to validate our findings externally and recommendations for incorporating more diverse data sources. We have mentioned that the ensemble method could alleviate the limitations of the base classifiers by leveraging their combined strengths, thus providing a more robust and reliable predictive performance.

By clarifying these limitations and potential biases, we aim to ensure that the readers fully understand the context and scope of our findings, alongside the inherent challenges and the measures taken to address them. This comprehensive exploration aids in establishing the reliability of our research and its implications for clinical practice.

Locations of Changes in the Revised Manuscript:

- Add: Ensemble Model Construction (Section 2.3.2, pages 14 and 15).
- Update: Results and discussion (Section 3).
- Update: Conclusions (Section 4, page 25).
- In clinical settings, the explainability of predictive models is crucial for user trust and adoption. The study could be improved by incorporating aspects of model explainability and interpretability, particularly in explaining decisions in critical care scenarios.

***Response****:*

Thank you for your valuable comment. In response, we recognize that in clinical settings, the explainability of predictive models is crucial for users. To address this, we have incorporated aspects of model explainability and interpretability, particularly in explaining decisions in critical care scenarios. This includes using techniques such as feature importance analysis provided by Random Forest (RF) and Gradient Boosting Machine (GBM) models.

First, we selected the most important features using RF and GBM. These feature importance scores helped us identify and rank the variables that significantly impact the model's predictions. By focusing on these critical features, we ensured that our models are both interpretable and efficient.

Next, we used these selected features to make predictions with our machine learning, deep learning, and ensemble learning models. By leveraging the most influential variables, we aimed to enhance the predictive performance of our models.

In the discussion section, we elaborate on how these selected features are effective in increasing the prediction accuracy of our models. We demonstrate that by focusing on the most important variables identified through RF and GBM, our models achieved improved accuracy and robustness. This detailed analysis helps in understanding the practical impact of feature selection on the performance of predictive models in critical care settings, ultimately supporting better decision-making processes.

By ensuring that our models are not only accurate but also interpretable, we aim to bridge the gap between complex predictive analytics and practical clinical utility, ultimately improving patient outcomes.

Locations of Changes in the Revised Manuscript:

- Update: Handling Missing Values and Applying Feature Importance (Section 2.2.1, page 8).
- Update: Results and discussion (Section 3, last paragraph).

# Comments from Reviewer 5:

The problem stated in this paper is relatively simple in domain of machine learning. I did not deny its importance in the field of medical domain, but such a classification/regression problem (given data of a patient, determine if he/she needs ventilator) has been well studied, and many advanced techniques can be used to address this problem. Applying traditional machine learning algorithm such LDA, Catboost and ANN is nothing new. More advanced research has Regarding the Simulated Annealing (SA) to improve the ML and DL models' performance. I think a more realistic way of doing hyper parameter is doing grid search. The claimed performance improvement is questionable given the size of the dataset used in this paper (1092 records in total). The difference between each method, and before and after using SA is minor. Plus, I think in medical domain, sensitivity is more important than other metrics. assigning ventilator to a patient who does not need it might be a waster of resource, but not assigning a ventilator to a patient who actually need it will be life threatening consequence. If we take a look at the sensitivity of all model results provided in this paper, it's around 50%, which is more like a random guessing given this is a binary classification problem (need or not need a ventilator).

***Response****:*

Thank you for your valuable feedback and for recognizing the importance of our work in the medical domain. We acknowledge your concerns and have addressed them in our revised manuscript as follows:

1. **Dataset Size and Accessibility**: We appreciate your concern regarding the size of our dataset. We agree that a larger dataset could potentially enhance the model's performance and robustness. However, acquiring extensive medical datasets involves issues of privacy and legal restrictions, as such datasets are not readily available or accessible due to confidentiality laws. Our dataset of 1,092 records is substantial within these constraints, and we have employed robust methods like ensemble learning and 10 fold cross validation to ensure the reliability of our findings despite the dataset's limited size. This limitation has been explicitly acknowledged and discussed in the discussion and conclusion sections of our manuscript.
2. **Model Selection and Innovation**: We incorporated XGBoost into our ensemble model due to its proven efficacy in various studies and its compatibility with our current classifiers—ANN, LDA, and CatBoost. While our dataset is mid-dimensional, we designed our ensemble to adeptly handle potential high-dimensional datasets in future applications. This strategic foresight ensures our model's adaptability and robustness across more complex data scenarios. XGBoost enhances the ensemble's capabilities by leveraging gradient boosting techniques, which are particularly effective in reducing bias and variance among predictions. The ensemble method capitalizes on the unique strengths of each algorithm, significantly improving overall model accuracy and reliability.
3. **Hyperparameter Optimization**: We have thoughtfully selected Simulated Annealing (SA) and Genetic Algorithms (GA) for hyperparameter tuning, rather than grid search. Considering the relatively high-dimensional nature of dataset in the medical domain, grid search would not only be computationally demanding but could also yield suboptimal results due to its exhaustive nature. SA and GA, inspired by natural processes, are exceptionally efficient at navigating complex search spaces. These methods excel in finding globally optimal solutions and adeptly avoid local optima, thus enhancing the effectiveness of our approach in managing the intricacies of larger and more complex hyperparameter spaces.
4. **Implementation of AutoML**: To further validate our approach, we implemented an AutoML framework to benchmark our ensemble model's performance against a state-of-the-art automated solution. This comparison not only reinforces the efficacy of our model but also provides a comprehensive analysis of their performance in contrast to automated approaches.
5. **Enhancing Sensitivity through Ensemble Modeling**: In addressing your concerns regarding the sensitivity of our predictive models, we have strategically implemented a weighted voting mechanism within our ensemble framework. This method has effectively improved the model’s sensitivity to 85%. By assigning different weights to the predictions from each individual model based on their performance and reliability, this approach fine-tunes our ensemble’s collective output. Such weighting is crucial in medical applications, as it significantly reduces the risk of false negatives. Additionally, this method leverages the strengths of each model to ensure that the most reliable and accurate predictions have a greater influence on the final decision, thus enhancing the overall predictive power and trustworthiness of the system in clinical settings.

Our revised manuscript includes detailed discussions and justifications for these choices, ensuring that our research approach is transparent and well-founded. We believe these modifications and clarifications will address your concerns and better demonstrate the robustness and relevance of our study.

We hope these revisions and explanations address your concerns and substantially strengthen our manuscript. We look forward to your feedback and are eager to make further modifications if required.

Thank you for your insightful critique, which has undoubtedly helped strengthen our paper.

# Comments from Reviewer 6:

Technically, I think this paper is well written. The algorithms and methodology are introduced clearly. I do have several problems from my perspective:

- I think we do need some intuition of using certain ML mythology. Table1 introduces some of the ML method that other paper were using. It seems you are using some method that others not used before. But that is not sufficient to demonstrate the reason us using CatBoost, LDA or ANN.

***Response****:*

Thank you for your positive feedback on the technical aspects of our paper and for highlighting the need for a more detailed explanation of our choice of ML methodologies, specifically CatBoost, LDA, ANN, and XGBoost. We appreciate your observation and have updated our manuscript to provide a clearer rationale for selecting these models.

In our selection process, we considered each model's unique capabilities and their compatibility with the specific requirements of our dataset and research objectives. We chose CatBoost for its ability to handle categorical features without extensive preprocessing and its efficiency in both regression and classification tasks. LDA was selected for its strength in dimensionality reduction and class distinction based on linear combinations of features, which is vital for medical datasets. ANN was chosen for its capability to model non-linear relationships in medical data through deep learning architectures. Also, XGBoost was included due to its proven efficacy in numerous studies and its recommendation by the TPOT AutoML algorithm, enhancing the ensemble's performance. These models were validated against empirical evidence, ensuring their superior performance in metrics such as Accuracy, Precision, Sensitivity, Specificity, and F1-score, as documented in [26] study.

Additionally, we reviewed related studies, as presented in Table 1 of the manuscript, to support our model selection. This ensures that each model contributes uniquely to the ensemble, enhancing the overall predictive power. The integration of XGBoost into our ensemble model provides a robust platform for comparative analysis against other classifiers, leveraging each model's strengths to enhance the ensemble's overall performance. This strategic choice is detailed in the revised manuscript, which explains the selection process and the reasons these models are well-suited for our specific data characteristics and research goals.

Locations of Changes in the Revised Manuscript:

•	Update: Machine Learning & Metaheuristic Models (Section 2.3.1, paragraph 2, page 11).

- If the focus for this paper is hyperparameter tuning, I think you need to explain why you choose such ranges for parameter tuning.

***Response****:*

Thank you for your feedback emphasizing the importance of hyperparameter range selection in our study. As outlined in revised manuscript, the selection of hyperparameter ranges for each ML and DL model is meticulously documented in Table 3. These ranges were not arbitrarily chosen; rather, they were established based on a blend of reliable sources to ensure optimal model performance tailored to our specific research needs.

Firstly, we utilized the official developer documentation for each model, which provides foundational guidelines on hyperparameter ranges that ensure the models operate within their designed capabilities. Additionally, we sought expert feedback tailored specifically to our research context. These insights helped refine the hyperparameter bounds to better suit the characteristics and challenges inherent in our dataset, such as variability, overfiting and feature distribution.

Moreover, we incorporated empirical evidence from related studies, as cited in the last column of Table 3. These references were instrumental in understanding how similar models were configured and optimized in comparable research scenarios, providing a validation for our choices.

This comprehensive approach in selecting hyperparameter ranges ensures that our tuning process is both theoretically sound and pragmatically validated, thus enhancing the reliability and applicability of our research findings.

Locations of Changes Related to this Comment in the Revised Manuscript:

- Machine Learning & Metaheuristic Models (Section 2.3.1, paragraph 4, pages 11 and 12).
- If we also want to compare between different models, we might want to make sure their search space are similar, or at least within the similar number of parameter combinations. It seems ANN's search space is bigger than other two. To this end, I think the comparison might not be fair.

***Response****:*

Thank you for your feedback. We appreciate the opportunity to clarify how we approached this in our study.

Due to the inherent differences in the nature of the machine learning algorithms employed—CatBoost, LDA, ANN, and XGBoost—each requires a distinct set of hyperparameters, which inevitably results in varying breadth and complexity of their respective search spaces. While CatBoost and LDA might have fewer hyperparameters, ANN and XGBoost typically requires a more extensive range of parameters to be tuned, especially to prevent overfitting and to optimize performance.

In addressing this, we ensured that the hyperparameter tuning for each model was as rigorous and comparable as possible within the constraints of their respective algorithms. This involved defining a broader range for some classifiers where necessary, based on both predefined settings recommended by the algorithm developers and values validated by other research studies.

Additionally, the complexity of ANN's implementation requires us to consider more constraints, such as the number of layers, units per layer, and loss functions, to ensure the generation of valid and reliable solutions by our metaheuristic algorithms. These constraints are crucial for a fair comparison as they help mitigate the risk of overfitting and ensure that each model operates within its optimal parameter range.

We believe that our methodological choices, although challenging, were necessary to provide a robust and fair comparative analysis of these models. In the revised manuscript, we have further detailed these considerations to ensure transparency and allow readers to fully understand the rationale behind our approach and the comparability of the results.

Locations of Changes Related to this Comment in the Revised Manuscript:

- Machine Learning & Metaheuristic Models (Section 2.3.1, paragraphs 3, page 11).
- There are many hyperparameter tuning methods. For example, AutoML works well if you want to compare between models. Can you compare the pros and cons of your method versus AutoML frameworks? In other words, why not using AutoML.

***Response****:*

Thank you for your feedback and for suggesting the potential use of AutoML in our study. We appreciate the opportunity to discuss the rationale behind our choice of hyperparameter tuning methods versus AutoML. In revised manuscript, we have indeed implemented AutoML as part of our comparative analysis. We compared the results obtained from TPOT AutoML, which primarily utilizes Genetic Algorithm (GA) for tuning, with those from our ensemble models, where the hyperparameters of the base classifiers were tuned using both Simulated Annealing (SA) and GA. This approach allowed us to directly evaluate the performance and efficacy of AutoML in relation to our more manually intensive, customized tuning processes.

The primary advantage of using both SA and GA in our study is the ability to have granular control over the tuning process, which is particularly beneficial for complex datasets and model configurations. These metaheuristic algorithms provide flexibility in exploring the hyperparameter space that AutoML may not offer, especially when dealing with specific constraints or preferences that are tailored to our research objectives. Furthermore, our predefined range of hyperparameters is broader than that typically utilized by TPOT AutoML, allowing for more extensive exploration and optimization. Conversely, AutoML offers a more streamlined and automated approach, reducing the need for detailed algorithmic knowledge and manual configuration. This makes AutoML highly accessible and efficient, particularly for general applications where extensive customization is not required. However, it can sometimes result in a lack of transparency in the model training process, which can be a limitation for research purposes where understanding the influence of each hyperparameter is crucial.

In the revised manuscript, we have highlighted these comparisons, detailing the pros and cons of using SA and GA versus AutoML frameworks. This includes discussions on the trade-offs between control and convenience, as well as the impact of each method on the overall performance and interpretability of our models. We believe this comprehensive analysis will provide readers with valuable insights into the appropriate contexts and conditions for the use of these different hyperparameter tuning approaches.

Locations of Changes Related to this Comment in the Revised Manuscript:

- Abstract.
- Comparison (Section 2.3.3, paragraphs 2, 3, and 4, pages 15, 16, and 17).
- Results and discussion (Section 3, paragraphs 6).
- Results and discussion (Section 3).
- Add: Table 7
